# Statin-induced anti-HMGCR myopathy: successful therapeutic strategies for corticosteroid-free remission in 55 patients

**DOI:** 10.1186/s13075-019-2093-6

**Published:** 2020-01-08

**Authors:** Alain Meyer, Yves Troyanov, Julie Drouin, Geneviève Oligny-Longpré, Océane Landon-Cardinal, Sabrina Hoa, Baptiste Hervier, Josiane Bourré-Tessier, Anne-Marie Mansour, Sara Hussein, Vincent Morin, Eric Rich, Jean-Richard Goulet, Sandra Chartrand, Marie Hudson, Jessica Nehme, Jean-Paul Makhzoum, Farah Zarka, Edith Villeneuve, Jean-Pierre Raynauld, Marianne Landry, Erin K. O’Ferrall, Jose Ferreira, Benjamin Ellezam, Jason Karamchandani, Sandrine Larue, Rami Massie, Catherine Isabelle, Isabelle Deschênes, Valérie Leclair, Hélène Couture, Ira N. Targoff, Marvin J. Fritzler, Jean-Luc Senécal

**Affiliations:** 1 Faculté de médecine, Université de Strasbourg, Service de rhumatologie et Centre de références des maladies autoimmunes rares, Hôpitaux universitaires de Strasbourg, Strasbourg, France; 2 Department of Medicine, Faculty of Medicine, University of Montreal, Montreal, Québec Canada; 3 Division of Rheumatology, Hôpital du Sacré-Coeur, Montreal, Québec Canada; 4 Division of Rheumatology, Centre hospitalier affilié universitaire régional de Trois-Rivières, Trois-Rivières, Québec Canada; 5 Division of Rheumatology, Centre Hospitalier de l’Université de Montréal (CHUM), 264, Boulevard René-Lévesque Est, Montréal, Québec H2X 1P1 Canada; 6 CHUM Research Center, Montréal, Québec Canada; 7 Service de médecine interne et immunologie clinique, Hôpital Pitié-Salpêtrière, Assistance publique Hôpitaux de Paris, Paris, France; 8 Division of Internal Medicine, Hôpital du Sacré-Coeur, Montréal, Québec Canada; 9 Faculty of Pharmacy, Laval University, Québec City, Québec Canada; 10 Division of Rheumatology, Hôpital Maisonneuve-Rosemont, Montréal, Québec Canada; 11 Department of Medicine, McGill University, Montreal, Canada; 12 Division of Rheumatology, Jewish General Hospital, Montreal, Canada; 13 Lady Davis Institute, Jewish General Hospital, Montreal, Canada; 14 Department of Neurology, McGill University, Montreal, Canada; 15 Montreal Neurological Institute and Hospital, Montreal, Canada; 16 Department of Pathology and Cell Biology, Faculty of Medicine, University of Montreal, Montreal, Canada; 17 Department of Pathology, Hôpital Maisonneuve-Rosemont, Montreal, Canada; 18 Department of Pathology, Hôpital Sainte-Justine, Montreal, Canada; 19 Department of Pathology, McGill University, Montreal Neurological Institute and Hospital, Montreal, Québec Canada; 20 Department of Medicine, Sherbrooke University, Sherbrooke, Québec Canada; 21 Division of Neurology, Hôpital Charles-Lemoyne, Longueuil, Canada; 22 Division of Rheumatology, Hôpital Charles-Lemoyne, Longueuil, Québec Canada; 23 Hôpital du Haut-Richelieu, Saint-Jean-sur-Richelieu, Québec Canada; 24 Departement of Medicine, Laval University, Québec City, Canada; 25 Centre Hospitalier Universitaire de Québec, Québec City, Québec Canada; 26 Veterans Affairs Medical Center, University of Oklahoma Health Sciences Center, Oklahoma City, USA; 27 Oklahoma Medical Research Foundation, Oklahoma City, OK USA; 28 Department of Medicine, Cumming School of Medicine, University of Calgary, Calgary, Alberta Canada

**Keywords:** Autoimmune myositis, Immune-mediated necrotizing myopathy, Anti-HMGCR myopathy, Statin, Therapy, Corticosteroid-free therapy, Immunosuppressant, IVIG, Remission

## Abstract

**Objective:**

To describe successful therapeutic strategies in statin-induced anti-HMGCR myopathy.

**Methods:**

Retrospective data from a cohort of 55 patients with statin-induced anti-HMGCR myopathy, sequentially stratified by the presence of proximal weakness, early remission, and corticosteroid and IVIG use at treatment induction, were analyzed for optimal successful induction and maintenance of remission strategies.

**Results:**

A total of 14 patients achieved remission with a corticosteroid-free induction strategy (25%). In 41 patients treated with corticosteroids, only 4 patients (10%) failed an initial triple steroid/IVIG/steroid-sparing immunosuppressant (SSI) induction strategy. Delay in treatment initiation was independently associated with lower odds of successful maintenance with immunosuppressant monotherapy (OR 0.92, 95% CI 0.85 to 0.97, *P* = 0.015). While 22 patients (40%) presented with normal strength, only 9 had normal strength at initiation of treatment.

**Conclusion:**

While corticosteroid-free treatment of anti-HMGCR myopathy is now a safe option in selected cases, initial triple steroid/IVIG/SSI was very efficacious in induction. Delays in treatment initiation and, as a corollary, delays in achieving remission decrease the odds of achieving successful maintenance with an SSI alone. Avoiding such delays, most notably in patients with normal strength, may reset the natural history of anti-HMGCR myopathy from a refractory entity to a treatable disease.

## Rheumatology key messages

- Anti-HMGCR myopathy with normal strength is common, and a corticosteroid-free induction strategy should be considered.
- In patients with proximal weakness, induction with corticosteroids + IVIG + a corticosteroid-sparing immunosuppressant is efficacious and may allow accelerated corticosteroid tapers.
- Targeting early remission increases the efficacy of a corticosteroid-sparing immunosuppressant regimen in maintaining remission.

## Introduction

Statin-induced immune-mediated necrotizing myopathy (IMNM) was initially described in patients on statin therapy who, despite statin discontinuation, developed a persistent myopathy, responsive only to immunosuppression [1]. It was later found that autoantibodies to 3-hydroxy-3-methyl-glutaryl-coenzyme A reductase (HMGCR) define that myopathy [2–4], that very high serum levels of creatine kinase (CK) and widespread damage on magnetic resonance imaging (MRI) are common [5], that sarcolemmal and capillary membrane attack complex (MAC) deposition are present on muscle biopsy [2, 6–8], and that intense immunosuppressive treatment is often needed [2, 7–12]. Recently, the pathogenicity of anti-HMGCR was demonstrated [13–15], and during the 224th European Neuromuscular Centre (ENMC) International Workshop [16], in the presence of proximal weakness and elevated CK levels, anti-HMGCR myopathy was defined. Compellingly, limb-girdle muscular dystrophy presentation [17] and isolated hyperCKemia [8] were reported as part of the spectrum of anti-HMGCR myopathy.

Therapeutically, efficacy of intravenous immunoglobulin (IVIG) monotherapy in statin-induced anti-HMGCR myopathy introduced the concept of corticosteroid-free induction strategy [18]. In parallel, a corticosteroid-based induction strategy composed of corticosteroids, IVIG, and a steroid-sparing immunosuppressant (SSI) was proposed as the initial treatment of severe anti-HMGCR myopathy [10]. Since this disease occurs in older patients who often have diabetes mellitus [19] and cardiovascular disease, corticosteroid-free induction and maintenance strategies are of utmost interest to minimize treatment-related morbidity [20, 21].

From a cohort of 55 patients, we studied the natural history and the spectrum of severity of untreated and treated statin-induced anti-HMGCR myopathy, while examining the therapeutic strategies that ultimately led to steroid-free remission.

## Methods

### Patients

The PHESEMO study (PHEnotype, SErology, and successful MOnotherapy maintenance in Autoimmune Myositis) is a retrospective study of patients with autoimmune myositis (AIM) followed longitudinally at Centre Hospitalier de l’Université de Montréal (CHUM) and Hôpital du Sacré-Coeur de Montréal (Montreal, QC, Canada) from 2001 to 2018. For the STATIN-PHESEMO study, only patients with statin-induced anti-HMGCR myopathy were considered, and additional patients from two University of Montreal affiliated hospitals (Centre Hospitalier Affilié Universitaire Régional de Trois-Rivières and Hôpital Maisonneuve-Rosemont) were included. The STATIN-PHESEMO study was approved by the CHUM Research Ethics Committee (reference number 2015-5607-CE14.248) and by the Research Ethics Committees of Hôpital du Sacré-Coeur (2014-1042), Centre Hospitalier Affilié Universitaire Régional de Trois-Rivières (2014-028-03), and Hôpital Maisonneuve-Rosemont (2015-639-CER14107).

The terminology of anti-HMGCR myopathy in this study refers only to patients with a statin-induced anti-HMGCR myopathy. Definite anti-HMGCR myopathy is defined as positive anti-HMGCR autoantibodies, elevated serum CK levels, and proximal skeletal muscle weakness (16). Probable anti-HMGCR myopathy was defined for this study as positive anti-HMGCR, elevated CK levels, suggestive muscle biopsy findings with necrosis/regeneration or MAC deposition, and normal strength. Possible anti-HMGCR myopathy was defined for this study as positive anti-HMGCR, elevated CK levels, and normal strength, irrespective of normal or absent muscle biopsy results.

### Data collection

Data on history, physical findings, and investigations were collected by retrospective medical record review using a standardized protocol. Data collection focused on demographics, myopathic features, chronology of events leading to the diagnosis (statin use, CKs, and clinical manifestations), treatment strategies (induction vs maintenance), and muscle biopsy findings.

### Definitions for therapy, remission, maintenance, and severity

These are shown in Additional file 1: Table S1.

### Identifying therapeutic subgroups within the STATIN-PHESEMO study

These are shown in Additional file 2: Table S2.

### Serology

Autoantibodies to HMGCR were detected by an addressable laser bead immunoassay (ALBIA) using a laboratory developed test (Mitogen Advanced Diagnostics, Calgary, AB, Canada) that was validated and then replaced by a commercially available ELISA (Inova Diagnostics, San Diego, CA, USA). Other AIM autoantibodies were detected by a commercial line immunoassay (Euroimmun GmbH, Lübeck, Germany) and included those directed to Jo-1, Mi2-α, Mi2-β, MDA5, NXP2, TIF1γ, PL7, PL12, PM/Scl75, PM/Scl100, Ku, SRP, EJ, OJ, and Ro52/TRIM21 autoantigens.

### Statistical analysis

Descriptive statistics were used to summarize the baseline characteristics of the study cohort. Continuous data were reported as medians with ranges, and categorical data were presented as counts with percentages.

To identify predictors of successful maintenance of remission with SSI monotherapy, we first used univariate logistic regression models to quantify the association between monotherapy maintenance and age, sex, CK, presence of normal strength and dysphagia at treatment initiation, and delay in treatment initiation, as well as use of corticosteroids and IVIG in induction. Then, a multivariate logistic regression model was done to identify independent predictors of monotherapy maintenance, incorporating variables that were significantly associated with monotherapy maintenance in univariate analyses. To account for potential residual confounding, we performed sensitivity analyses which additionally adjusted for omitted variables.

## Results

### Clinical characteristics of 55 patients with anti-HMGCR myopathy

Table 1 details baseline characteristics: the median age at diagnosis was 67.7 years, 95% were Caucasians, 72% had diabetes mellitus, and none had cancer within 3 years of diagnosis. The statin most commonly prescribed was atorvastatin (84%). A total of 22 patients (40%) presented with normal strength and elevated CK levels. Statin was discontinued in every patient.

**Table 1:** Baseline characteristics of patients with anti-HMGCR myopathy (*N* = 55)

	*n* (%) or median (range)
Sex, male/female	30/25
Age at diagnosis, median (range), years	67.7 (44–86.1)
Prior statin use, *n* (%)	55 (100)
Atorvastatin use, *n* (%)	46 (84)
Diabetes mellitus, *n* (%)	39 (72)
Cardiovascular disease, *n* (%)*	29 (53)
Cancer within 3 years of diagnosis, *n*	0
First serum CK levels at presentation, median (range), UI/L	2935 (500–19,465)
CK levels at treatment initiation, median (range), UI/L	5000 (554–23,000)
Myalgias, *n* (%)	21 (38)
Subjective oropharyngeal dysphagia, *n* (%)	16 (29)
Objective oropharyngeal dysphagia, *n* (%)	5 (9)
Proximal muscle weakness at presentation, *n* (%)	33 (60)
Proximal muscle weakness at treatment initiation, *n* (%)	46 (84)
Muscle biopsy, *n* (%)	54 (98)
Necrosis and regeneration, *n* (%)	48 (87)
Isolated sarcolemmal/capillary MAC deposition, *n* (%)	4 (7)
Regeneration only, *n* (%)	1 (2)
Normal, *n* (%)	1 (1)
Other abnormalities	
MHC-1 expression on non-necrotic muscle fibers, *n* (%)	26/51 (51)
Sarcolemmal and/or capillary MAC deposition, *n* (%)**	38/42 (90)

*Myocardial infarction or stroke

**MAC deposition was found on non-necrotic fibers and/or endomysial capillaries

At treatment initiation, 46 patients (84%) had proximal weakness, the median CK elevation was 5000 UI/L (range 554–23,000), 48 patients had biopsy evidence of a necrotizing myopathy, and all were positive for anti-HMGCR autoantibodies. Eighty-four percent (46/55) of patients had definite, 13% (7/55) probable, and 3% (2/55) possible anti-HMGCR myopathy.

### Corticosteroid-free induction strategies were successful in all 14 selected patients

The chronology of events leading to the initiation of treatment is detailed in Additional file 3: Table S3. Extensive delay between presentation and treatment was seen in 2 patients (57 and 78 months); interestingly, on statin discontinuation, CK levels had fallen under 500 UI/L, but ultimately rose to > 2100 U/L, leading to treatment.

**Fig. 1:**
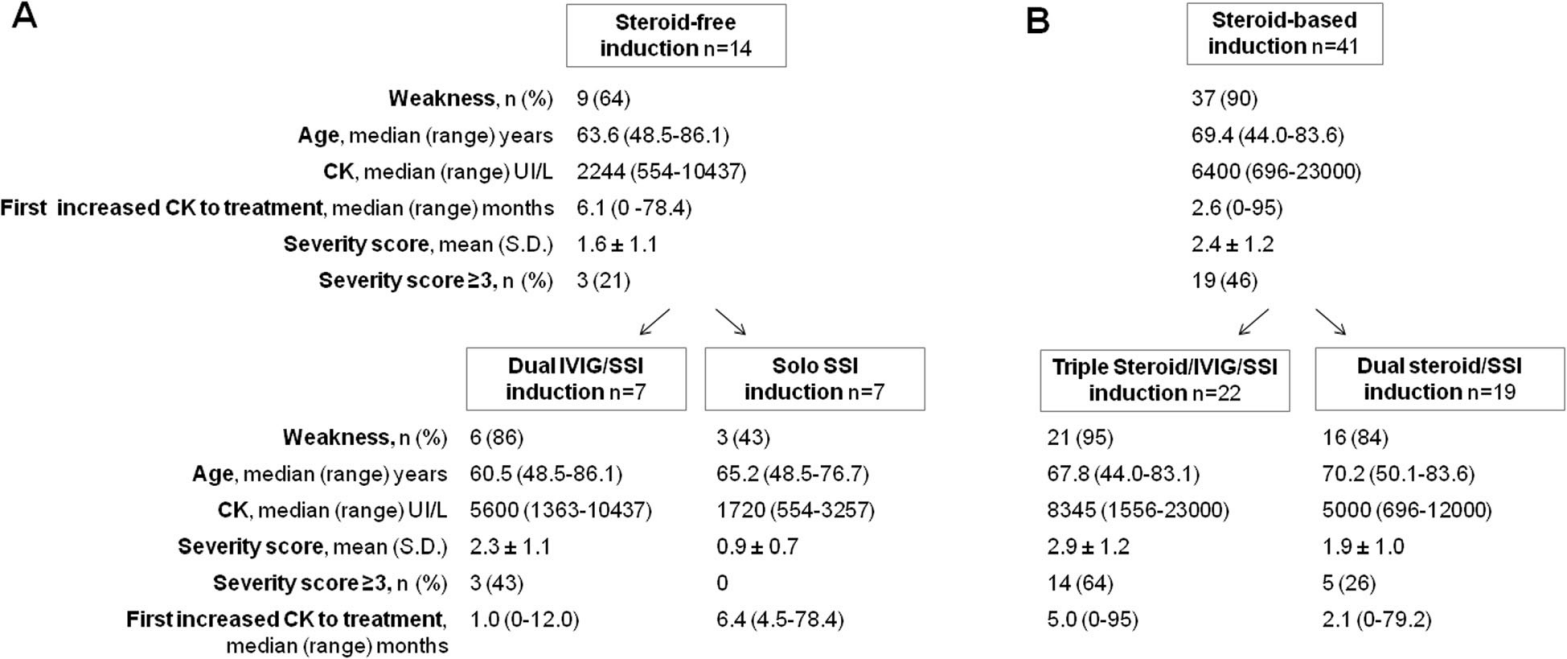
Flow diagram of corticosteroid-free induction therapy of 14 patients (**a**) and steroid-based induction therapy of 41 patients (**b**) with anti-HMGCR myopathy treated with a steroid-sparing immunosuppressant, with or without intravenous immunoglobulins

As shown in Fig. 1, the corticosteroid-free cohort consisted of 14 patients with a successful induction. Initial induction strategies were SSI monotherapy (*n* = 7) and Dual IVIG/SSI monotherapy (*n* = 7). As illustrated in Additional file 4: Table S4, the successful induction strategies were as follows: SSI monotherapy (*n* = 6 patients), SSI combination (*n* = 1), Dual IVIG/SSI monotherapy (*n* = 4), and Dual IVIG/SSI combination (*n* = 3). All evaluable maintenance strategies (*n* = 12) were successful.

The Solo SSI cohort included 7 patients with a median CK level of 1720 UI/L (range 554–3257), 3 (43%) of whom had proximal weakness but none considered severe (Fig. 1). As seen in Additional file 4: Table S4, induction with MTX alone was successful in 6 patients, with time to remission ranging from 1.9 to 34 months. An AZA/MTX step-up strategy was successful in 1 patient (no. 6) who had failed initial AZA induction monotherapy.

The Dual IVIG/SSI cohort included 7 patients with a median CK level of 5600 UI/L (range 1363–10,437), 6 (86%) of whom had proximal weakness and 3 (43%) with severe myopathy (Fig. 1). Initial induction with MTX/IVIG was successful in 4 patients, with time to remission ranging from 1 to 5 months. For the remaining 3 patients, successful step-up induction strategies of MTX/AZA/IVIG were needed.

### Corticosteroid-based induction strategies were successful in 41 patients

As shown in Fig. 1b and Table 2, the corticosteroid-based induction cohort consisted of 41 patients. Initial induction strategies were Dual steroid/SSI monotherapy (*n* = 19) and Triple steroid/IVIG/SSI monotherapy (*n* = 22). Adequate induction corticosteroid therapy was given in 36 (88%) patients. The decision to include IVIG in the induction strategy was left to the treating physician and was determined by the perceived severity of disease (confounded by indication) or failure of a Dual steroid/SSI induction strategy (*n* = 3 patients). Proximal weakness was present in 37 (90%) patients, and 19 (46%) had severe myopathy. Serum CK elevation ranged broadly from 696 to 23,000 UI/L. Delay before treatment initiation was also wide, ranging from immediate treatment to 95 months.

**Table 2:** Severity factors in patients with anti-HMGCR myopathy and successful steroid-based induction therapy, stratified by concomitant use of IVIG therapy and early vs late remission (*N* = 41)

	Dual steroids/SSI cohort (*n* = 19)		Triple steroids/IVIG/SSI cohort (*n* = 22)	
	Early remission (≤ 3 months), *n* = 10	Late remission (> 3 months), *n* = 9	Early remission (≤ 3 months), *n* = 12	Late remission (> 3 months), *n* = 10
Weakness at treatment onset, *n* (%)	8 (80)	8 (89)	11 (92)	10 (100)
Age at treatment onset, median (range) years	70.6 (59.9–83.6)	69.4 (50.1–81.3)	73.6 (46.5–83.1)	60.4 (44.0–74.3)
CK level at treatment onset, median (range) UI/L	2673 (696–12,000)	6405 (3573–10,465)	7317 (1556–13,339)	10,789 (2267–23,000)
Severity score, mean (SD)	1.4 (0.8)	2.5 (0.9)	2.5 (1.2)	3.3 (0.9)
Severity score ≥ 3, *n* (%)	1 (10)	4 (44)	6 (50)	8 (80)
Delay from first increased serum CK (> 500 UI/L) to treatment, median (range) months	1.4 (0–79.2)	13.4 (0–24.9)	0.8 (0–42.2)	11.5 (0–95)
Delay from treatment to serum CK < 500 UI/L, median (range) months	1.7 (0.4–3)	11.5 (4–50.7)	2.0 (0.6–3)	15.0 (3.2–53)
Needed induction strategies to obtain remission, *n* (%)				
1	10 (100)	8 (89)	12 (100)	6 (60)
2	0	1 (11)	0	1 (10)
3	0	0	0	2 (20)
≥ 4	0	0	0	1 (10)
Successful maintenance with SSI monotherapy, *n* (%)	8 (80)	8 (89)	5 (42)	2 (20)
Corticosteroid dosage at last follow-up				
No corticosteroids	8 (80)	8 (89)	9 (75)	8 (80)
Prednisone ≤ 5 mg per day	2 (20)	1 (11)	1 (8)	1 (10)
Prednisone > 5 mg per day	0	0	2 (17)	1 (10)
Drug-free remission, *n* (%)	3 (30)	1 (11)	0	0
Normal strength at last follow-up, *n* (%)	9 (90)	8 (89)	6 (50)	5 (50)

*SSI* steroid-sparing immunosuppressant

All corticosteroid-based induction strategies (*n* = 41) were successful. As seen in Fig. 1 and Table 2, patients were stratified first for initial IVIG use and then for early or late remission. Overall, only 52.6% (*n* = 10) of patients in the Dual steroid/SSI cohort and 54.5% (*n* = 12) of patients in the Triple steroid/IVIG/SSI cohort had an early remission. Importantly, patients with an early remission had a shorter median delay from presentation to treatment compared to patients with late remission (1.07 vs 12 months, *P* = 0.043).

Patients with a late remission (*n* = 9) in the Dual steroid/SSI cohort were analyzed (Table 2 and data not shown). Adequate induction corticosteroid therapy was given in all but 2 patients. Optimizing SSI therapy to achieve remission was frequent, as efficacious doses of MTX were 20–30 mg/week (*n* = 7), MMF 3 g/day (*n* = 1), and AZA/ALLO (*n* = 1). The initial induction strategy was successful in 8 patients, with a median time to remission of 7 months (range 4–22 months). An AZA/ALLO switching induction strategy was successful in 1 patient who had failed initial steroid/MTX induction therapy.

Patients with a late remission (*n* = 10) in the Triple steroid/IVIG/SSI cohort were also analyzed. Adequate induction corticosteroid therapy was given in 8 patients. For 3 patients with successful initial induction strategy, time to remission was between 4 and 5 months. For 3 additional patients, initial induction strategy was successful only when IVIG therapy was added. Time to remission of the latter patients was 13–18 months, but the late addition of IVIG therapy resulted in remission in ≤ 3 months. Failure of a Triple steroid/IVIG/SSI induction strategy was seen in the last 4 patients, and they had by definition refractory anti-HMGCR myopathy.

Induction strategies used in refractory anti-HMGCR (*n* = 4) were switching (*n* = 1) or step-up (*n* = 3). One patient achieved remission with an AZA/ALLO switching induction strategy, while 3 patients had successful step-up induction strategy with MTX/AZA, MTX/RTX, and MMF/ABA, respectively. For the 4 refractory patients, time to remission from the initial induction strategy was 8, 18, 18, and 53 months, respectively.

### Corticosteroid-free maintenance was successful in 73% of patients treated at induction with corticosteroids

As shown in Table 3, a corticosteroid-free SSI monotherapy maintenance strategy was successful in 22 patients, while a SSI monotherapy maintenance with daily prednisone ≤ 5 mg was effective in one patient. Normal strength at last follow-up was seen in 87% (*n* = 20/23) of patients. Drug-free remission was possible in 4 patients.

**Table 3:** Severity factors for successful steroid-free maintenance therapy in patients with anti-HMGCR myopathy stratified by the use of steroid-sparing immunosuppressants in monotherapy or in combination, with or without IVIG (*N* = 41)

	Successful maintenance with SSI monotherapy, *n* = 23	Unsuccessful or unevaluable maintenance with SSI monotherapy				
		Overall, *n* = 18	Remission with SSI monotherapy and IVIG, *n* = 5	Remission with SSI combination therapy (± IVIG), *n* = 5	Unsuccessful maintenance with SSI therapies, *n* = 6*	Maintenance with SSI therapy not evaluable, *n* = 2
Weakness at treatment onset, *n* (%)	21 (91)	16 (89)	5 (100)	5 (100)	5 (83)	1 (50)
Age at treatment onset, median (range) years	70.5 (50.1–83.6)	67.5 (44.0–83.1)	69.4 (56.7–78.4)	66.2 (44.0–78.8)	63.0 (46.5–74.8)	67.5 (73.0–83.1)
CK at treatment onset, median (range) UI/L	5380 (696–23,000)	8234 (1556–14,098)	4750 (2770–14,098)	8300 (1556–11,755)	6737 (2267–13,339)	6327 (2832–9821)
Severity score, mean (SD)	2.2 ± 1.1	2.7 ± 1.2	2.8 ± 0.8	3.4 ± 0.9	2.5 ± 1.4	1.5 ± 2.1
Severity score ≥ 3, n (%)	8 (35)	11 (61)	3 (60)	4 (80)	3 (50)	1 (50)
Delay from first increased serum CK (< 500 UI/L) to treatment, median (range) months	1.7 (0–24.9)	12.7 (0–95.0)	14 (0–95.0)	13.4 (0.4–26.0)	6.6 (0–42.2)	0 and 79.0
IVIG at last follow-up	0	11 (61)	5 (100)	2 (40)	3 (50)	1 (50)
Corticosteroid dosage at last follow-up						
No corticosteroids	22 (96)	11 (61)	4 (80)	4 (80)	3 (50)	0
Prednisone ≤ 5 mg per day	1 (4)	4 (22)	1 (20)	1 (20)	1 (17)	1 (50)
Prednisone > 5 mg per day	0	3 (17)	0	0	2 (33)	1 (50)
Drug-free remission, *n* (%)	4 (17)	0	0	0	0	0
Normal strength at last follow-up, *n* (%)	20 (87)	8 (44)	2 (40)	3 (60)	2 (33)	1 (50)

*SSI* steroid-sparing immunosuppressant

*Unsuccessful maintenance with SSI therapy included failure to SSI monotherapy (*n* = 3), SSI monotherapy + IVIG (*n* = 2) and SSI combination therapy + IVIG (*n* = 1)

In the remaining 18 patients not meeting the definition of successful maintenance strategy with SSI monotherapy, 8 patients had a steroid-free successful maintenance strategy: 4 patients had a maintenance strategy of SSI monotherapy plus IVIG whereas 4 patients had a maintenance strategy of an SSI combination (with or without IVIG) (Table 3). Overall, 73% (*n* = 30/41) of patients treated with corticosteroids at induction had a successful steroid-free maintenance.

In this corticosteroid-based induction cohort, the SSIs used for successful maintenance, alone or in combination, were MTX (*n* = 25), AZA (*n* = 3), AZA/ALLO (*n* = 2), MMF (*n* = 7), and ABA (*n* = 1). SSI combinations used successfully (with or without IVIG) were MTX/AZA (*n* = 2), MTX/MMF (*n* = 2), and MMF/ABA (*n* = 1). The median follow-up was 52 months (range 14–140 months).

### Early diagnosis of anti-HMGCR myopathy with hyperCKemia but normal strength favored corticosteroid-free induction strategies

**Fig. 2:**
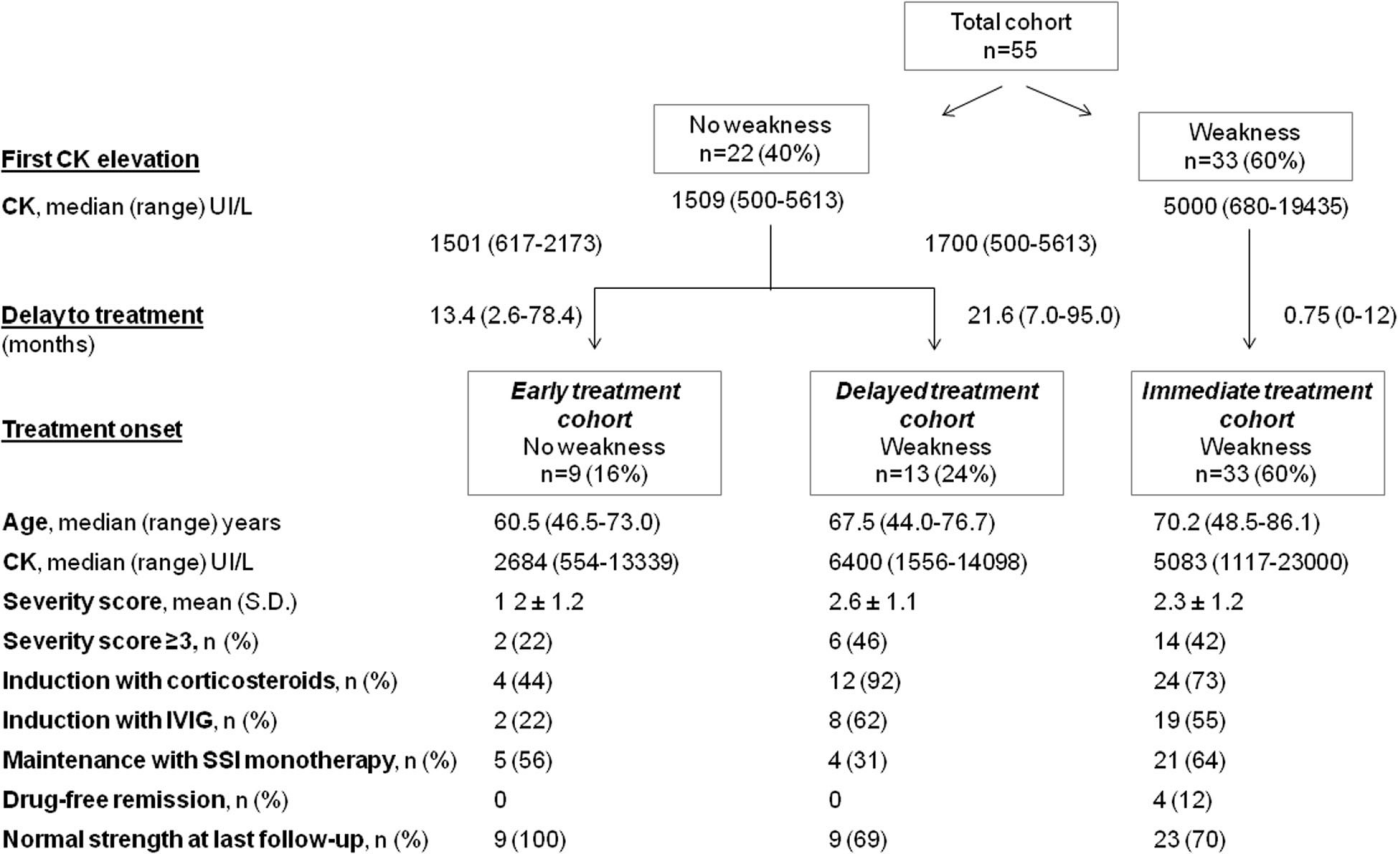
Flow diagram of induction and maintenance therapies of 55 patients with anti-HMGCR myopathy stratified by the presence of proximal weakness, both at disease presentation and initiation of treatment

As shown in Fig. 2, 22/55 (40%) patients had no weakness at presentation and initiation of treatment, and hyperCKemia was the first manifestation of anti-HMGCR myopathy. The median CK level at presentation of these 22 patients was 1509 UI/L (range 500–5613 UI/L).

Figure 2 also shows that corticosteroid-free induction was used in 56% (*n* = 5/9) in the early treatment cohort, versus 8% (*n* = 1/13) in the delayed treatment cohort (*P* = 0.023), indicating that patients with anti-HMGCR myopathy with normal strength were candidates for corticosteroid-free induction.

The chronology of events leading to treatment in these 22 patients is detailed in Additional file 5: Table S5. Interestingly, after statin discontinuation, serum CK levels dropped by ≥ 50% in 6 (27%) of these 22 patients.

### Early treatment of anti-HMGCR myopathy increased the efficacy of SSI monotherapy in maintaining remission

The first evidence of a long-term benefit of treating anti-HMGCR myopathy early, i.e., successful corticosteroid-free SSI monotherapy maintenance, is illustrated in Table 3. The median delays in initiating treatment in patients with successful SSI monotherapy maintenance (*n* = 23) vs those without (*n* = 18) were 1.7 month and 12.7 months, respectively (*P* = 0.048), in favor of early intervention.

The second evidence is shown in Fig. 2. The rate of successful remission maintenance with SSI monotherapy was 64% in the immediate treatment cohort as opposed to only 31% (*P* = 0.056) in the delayed treatment cohort (median delay to treatment 0.75 vs 21.6 months, respectively).

Table 4 presents statistical analyses of predictive factors for successful SSI monotherapy maintenance. Delay in treatment initiation was independently associated with a lower odds of successful maintenance with an SSI monotherapy (OR 0.92, 95% CI 0.85 to 0.97, *P* = 0.015). In addition, IVIG use in induction was strongly and significantly associated with a reduced odds of successful monotherapy maintenance (OR 0.08, 95% CI 0.01 to 0.32, *P* = 0.001). Sensitivity analyses additionally adjusted for age, sex, strength, CK, dysphagia, and corticosteroid use were consistent with these results.

**Table 4:** Univariate and multivariate analyses of predictive factors for successful maintenance with steroid-sparing immunosuppressant monotherapy in patients with anti-HMGCR myopathy (*N* = 55)

	Maintenance of remission with SSI monotherapy		Univariate	Multivariate
	Yes (*n* = 30), *n* (%) or Median (range)	No (*n* = 25), *n* (%) or Median (range)	OR (95% CI), *P* value	OR (95% CI), *P* value
Age at treatment initiation, Median (range) years	68.2 (48.5–86.1)	66.9 (44.0–83.1)	1.02 (0.97 to 1.08), *P* = 0.516	–
Male sex, *n* (%)	17 (57)	13 (52)	1.21 (0.41 to 3.54), *P* = 0.729	–
Normal strength at treatment initiation, *n* (%)	5 (17)	4 (16)	1.05 (0.25 to 4.72), *P* = 0.947	–
CK at treatment initiation, Median (range) UI/L	4959 (554–23,000)	5083 (1533–14,098)	1.00 (1.00 to 1.00), *P* = 0.582	–
Dysphagia, subjective, *n* (%)	9 (30)	7 (28)	1.10 (0.34 to 3.65), *P* = 0.871	–
Dysphagia, objective, *n* (%)	1 (3)	4 (16)	0.18 (0.01 to 1.33), *P* = 0.139	–
Delay between first increased serum CK and treatment initiation, Median (range) months	2.0 (0–24.9)	11.0 (0–95.0)	0.94 (0.88 to 0.98), *P* = 0.025	0.92 (0.85 to 0.97), *P* = 0.015
Use of corticosteroids in induction, *n* (%)	23 (77)	18 (72)	1.28 (0.37 to 4.39), *P =* 0.693	–
Use of IVIG in induction, *n* (%)	10 (33)	19 (76)	0.16 (0.04 to 0.50), *P* = 0.002	0.08 (0.01 to 0.32), *P* = 0.001

*SSI* steroid-sparing immunosuppressant

## Discussion

This case series provides an overview of the disease spectrum of statin-induced anti-HMGCR myopathy, ranging from presentation as an acute IMNM [2] to persistent hyperCKemia despite statin discontinuation. The initial 12 patients from the present cohort were described previously [8], and thereon, access to anti-HMGCR autoantibody testing allowed diagnosis of anti-HMGCR myopathy in 43 additional patients.

The initial description of 8 patients with a progressive, MHC-I positive myopathy associated with statin therapy was noteworthy for their complete response to MTX and prednisone [1]. Subsequent reports demonstrated that anti-HMGCR myopathy was difficult to treat [7–10] and that younger patients were harder to treat than older patients [11].

There is no uniform approach to the treatment of anti-HMGCR myopathy [16, 22–24], nor are there a described severity score [2] or treat to target recommendations [25]. The 224th ENMC definition of severe anti-HMGCR myopathy was the presence of walking difficulties and/or dysphagia, while partial remission was defined as an improvement ≥ 110% of MMT-8 and/or CK levels, the latter remaining greater than or equal to twice the normal range, i.e., ≥ 500 UI/L [16]. The definition of complete remission consisted of normal strength and normal serum CK levels [16].

Achieving sustained remission with normal CK levels, normal strength, and no corticosteroids is indeed a goal of treatment. But both steroid myopathy and MRI-documented damage may occur [4], and remission may be present without full recovery of strength. In a large cohort of treated anti-HMGCR myopathy, strength recovery was often seen with persistent serum CK elevation > 500 UI/L, a sign of ongoing activity [11]. In another study, CK levels were shown to be closely associated with disease activity [25]. In the present cohort of 55 patients, hyperCKemia ≥ 500 UI/L with normal strength was the presentation of 40% of patients. Taken altogether, these results suggest that in anti-HMGCR myopathy, achieving a serum CK level ≤ 500 UI/L could define remission and be the goal of both successful induction and maintenance strategies. Targeting early remission may be justified to minimize steroid therapy [21]. Indeed, IVIG use in the 3 months after presentation of necrotizing myopathy was associated with better outcomes at 6 months [10].

In the present study, analysis of the corticosteroid-free induction cohort highlighted the relative contributions of IVIG and an SSI in an induction strategy. Patients with successful Solo SSI strategy achieved remission up to 13 months after initiation of treatment. In contrast, patients with a successful Dual IVIG/SSI strategy often achieved remission within 3 months, illustrating the efficacy of IVIG. Steroid-free induction strategies were demonstrated to be efficacious, thus sparing steroid toxicity in an older population with diabetes and cardiovascular disease. As shown by Mammen and Tiniakou, further studies will identify ideal candidates for steroid-free induction of anti-HMGCR myopathy [18].

Analysis of the corticosteroid-based induction cohort confirmed that a Triple IVIG/steroid/SSI induction was efficacious in most patients since only 4/22 (18%) patients failed that induction strategy. As for treating anti-HMGCR myopathy with a Dual steroid/SSI strategy, late remissions were frequent, inferring that early optimization of SSI is essential and corticosteroids less efficacious than previously thought. The 224th ENMC recommended prednisone 1 mg/kg/day [16] for treatment of severe anti-HMGCR myopathy. However, tapering corticosteroids only when CK levels have normalized, as recommended in general for AIM treatment [26, 27], may not apply to anti-HMGCR myopathy. Indeed, if IVIG and an optimized SSI are introduced as the initial treatment, it is possible to tailor initial steroid dosing to both comorbidities and disease severity, and the promptness of the corticosteroid taper to the early CK response, therefore tapering corticosteroids even when CK levels have not yet normalized.

In this study, the corticosteroid-based induction strategy achieved steroid-free remission in 73%, normal strength at last follow-up in 68%, and steroid-free successful maintenance with SSI monotherapy in 54% of patients. In contrast, the corticosteroid-free induction strategy achieved steroid-free remission in 100%, normal strength at last follow-up in 93%, and successful maintenance with an SSI monotherapy in 50% of patients. Successful, steroid-free maintenance with either SSI monotherapy with IVIG, or combination SSI therapy (with or without IVIG) was observed in 20% of the steroid-based cohort and in 29% of the steroid-free cohort. Overall, these results argue that sustained remission without corticosteroids is possible in anti-HMGCR myopathy.

Increasing the use of various SSI combinations in induction for refractory patients, but especially in maintenance strategies, may ultimately allow successful steroid-free and IVIG-free maintenance therapy. The successful strategies used in refractory and relapsing patients were either to switch to another SSI or to add an additional SSI through a step-up strategy.

A striking 40% of patients (*n* = 22/55) presented initially with persistent hyperCKemia and normal strength. Statin discontinuation led to a ≥ 50% drop in CK levels in 27–31% of patients, hinting to a natural history-based window of opportunity for successful early treatment. Although some patients were treated years later while they still had normal strength, it is noteworthy that proximal weakness ensued in many patients, with a median delay to treatment of 21.6 months. The near-ineluctability of progressive myopathy in patients presenting with hyperCKemia is one argument for early treatment. Another convincing argument for early treatment would be if accrual damage in untreated disease would hasten the appearance of refractory disease. In untreated anti-HMGCR myopathy, regenerating muscle cells express high levels of HMGCR, sustaining and perhaps intensifying the autoimmune response with time, even after statins are discontinued [3].

Multivariate analysis revealed a crucial therapeutic finding, namely that delay in initiating treatment, even with hyperCKemia alone, decreases the probability of a successful SSI monotherapy maintenance. Indeed, refractory anti-HMGCR myopathy is frequent in patients with limb-girdle muscular dystrophy-like presentation [17], illustrating the consequence of delaying treatment. Moreover, early treatment might offer hope for drug-free remission, as achieved by 4 patients in the immediate treatment induction cohort. Weighting the advantages of safer, steroid-free induction against the consequences of delaying remission and missing a window of opportunity should be analyzed in future studies.

Limitations of the present study are a retrospective design, the lack of a standardized therapeutic approach, and the absence of documentation of corticosteroid toxicity. A strength of this study is the careful analysis of treatment strategies in 55 patients representing the full spectrum of statin-induced anti-HMGCR myopathy. Another strength is the long follow-up that allowed analysis of both induction and maintenance strategies, leading to the suggestion that early treatment with IVIG plus an SSI, with or without corticosteroids, is appropriate in most patients. Indubitably, randomized trials of IVIG-based initial treatment strategies in statin-induced anti-HMGCR myopathy are needed, leading to individualized treatment tailored to disease severity.

## Conclusion

In summary, the present study convincingly expanded the spectrum of anti-HMGCR myopathy to include isolated hyperCKemia, demonstrated the efficacy of steroid-free induction strategies in selected patients, validated the proposed Triple steroid/IVIG/SSI induction strategy, and confirmed that steroid-free maintenance is an achievable goal, occasionally through the use of SSI combinations or an SSI/IVIG maintenance. Finally, avoiding delays in treatment, most notably in patients with normal strength, may reset the natural history of anti-HMGCR myopathy from a refractory entity to a treatable disease.

## Supplementary information

**Additional file 1 : Table S1.** Definitions for therapy, remission, maintenance and severity of anti-HMGCR myopathy.

**Additional file 2 : Table S2**. Identifying therapeutic subgroups within the STATIN-PHESEMO study.

**Additional file 3 : Table S3.** Chronology of events leading to corticosteroid-free treatment of patients with anti-HMGCR myopathy (*N* = 14).

**Additional file 4 : Table S4.** Corticosteroid-free induction and maintenance therapy of patients with anti-HMGCR myopathy (*N* = 14)

**Additional file 5 : Table S5.** Chronology of events, from disease onset to maintenance therapy, in patients with anti-HMGCR myopathy presenting with normal strength (*N* = 22)

## Data Availability

Anonymized data not published within the article will be shared upon request from any qualified investigator.

## Abbreviations

ABA: Abatacept
ALLO: Allopurinol
AZA: Azathioprine
CK: Creatine kinase
ENMC: European Neuromuscular Centre
HMGCR: 3-Hydroxy-3-methyl-glutaryl-coenzyme A reductase
IVIG: Intravenous immunoglobulins
MMF: Mycophenolate mofetil
MAC: Membrane attack complex
MRI: Magnetic resonance imaging
MTX: Methotrexate
RTX: Rituximab
SSI: Steroid-sparing immunosuppressant
