# Supplementary material for: Statin-induced anti-HMGCR myopathy: successful therapeutic strategies for corticosteroid-free remission in 55 patients
Source: Arthritis Res Ther. 2020 Jan 8;22:5. doi: 10.1186/s13075-019-2093-6 (PMC6950801; doi:10.1186/s13075-019-2093-6)
Supplement: Supplementary file 1 — Additional file 1 : Table S1. Definitions for therapy, remission, maintenance and severity of anti-HMGCR myopathy. [file 13075_2019_2093_MOESM1_ESM.docx]

**Supplementary Table S1**

**Definitions for therapy, remission, maintenance and severity of anti-HMGCR myopathy**

**Adequate induction corticosteroid therapy**: use of a daily prednisone dose of at least 50 mg during at least 1 month, followed by gradual taper, or use of weekly intravenous methylprednisolone pulses (500 mg) during at least one month, with spacing of pulses nearing remission.

**Corticoresistance**: the inability of adequate induction corticosteroid therapy alone to induce remission. Historical expert recommendation of corticosteroid therapy in myositis [27, 28] is an initial dose of 60 to 80 mg/day, with taper initiated after 4 to 8 weeks, or until serum muscle enzymes normalize. For the purpose of this study, where a concomitant SSI agent was routinely used at induction, the inability of adequate induction corticosteroid therapy to induce an early remission, i.e ≤ 3 months, was considered as corticoresistance. As a corollary, late remission, i.e. ≥ 3 months on a dual corticosteroid/SSI induction strategy, may illustrate both the delayed efficacy of SSI for inducing remission and the minor contribution of steroids, in reaching remission.

**Steroid-sparing immunosuppressants**: SSI medications included methotrexate (MTX), azathioprine (AZA), AZA combined with allopurinol (AZA/ALLO) when unfavourable AZA metabolism is present, mycophenolate mofetil (MMF), cyclosporine A (CsA), tacrolimus (TACRO) or a biologic agent such as rituximab (RITUX), abatacept (ABA) or etanercept (ETA).

**Myositis diagnosis date**: the date on which treatment is initiated.

**Induction**: the time period during which a treatment is used to induce remission.

**Induction strategy**: the therapeutic treatment leading to remission. For the purpose of the study, failed therapy with dual corticosteroid/SSI induction followed by a successful rescue with IVIG is considered as a single induction strategy. Achieving remission is the target of an induction strategy.

**Remission**: the date on which the serum CK level falls below 500 U/L with respect to the induction treatment; an **early** remission is ≤ 3 months, a **late** remission is ≥ 3 months. Normal strength with CK levels ≥ 500 IU/L is not considered a remission. Achieving remission is the target of an induction strategy.

**Maintenance**: the time period when a therapeutic strategy is used to maintain remission. Maintenance begins when remission is achieved, yet an SSI may take 3 to 6 months to be efficacious, and thus to be evaluable.

**Maintenance strategy**: the therapeutic regimen leading to successful maintenance treatment. For this study, the first successful maintenance strategy without IVIG is chosen. However, if anti-HMGCR myopathy flared on IVIG discontinuation, then the successful maintenance strategy with IVIG is chosen. A successful maintenance strategy is defined as remission for 1 year with no corticosteroids or ≤ 5mg of daily prednisone. Maintaining steroid-free remission, or with daily prednisone of ≤ 5mg, for 1 year is the target of maintenance strategy in this study.

**Step-up induction strategy**: adding a second SSI to an initial corticosteroid-free or corticosteroid-based failed induction strategy; failed dual corticosteroid/SSI induction with a successful rescue with IVIG is not considered as a step-up induction strategy. Achieving remission is the target of a step-up strategy.

**Step-up maintenance strategy**: adding an SSI or IVIG to a failed initial maintenance strategy. Maintaining remission is the target of a step-up maintenance strategy.

**Switching induction strategy**: replacing one SSI by another in any failed initial induction strategy. Achieving remission is the target of a switching induction strategy.

**Switching maintenance strategy**: replacing one SSI by another in any failed initial maintenance strategy. Maintaining remission is the target of a switching maintenance strategy.

**Efficacious vs failed strategy**: A strategy is deemed efficacious based on its ability to induce or maintain remission. A failed strategy is defined by its inability to induce or maintain remission; early discontinuation of an SSI due to a side effect is not considered a failed strategy.

**Refractory anti-HMGCR myopathy**: the inability of adequate induction corticosteroid therapy plus IVIG plus a SSI to induce remission.

**Optimizing steroid-sparing immunosuppressant therapy**: for MTX and MMF, aiming for higher doses; for AZA, screening for the presence of unfavourable metabolism and adding allopurinol.

**Drug-free remission**: a corticosteroid-free patient who is still in remission after 18 months of cessation of SSI therapy.

**Severity score**

This descriptive score is composed of putative severity factors in anti-HMGCR myopathy. The aim of the score is to reflect retrospectively the perceived severity of disease by the treating physician in order to illustrate better the chosen induction strategy. The putative risk factors were:

- age under 65 years = 1 point [11]
- proximal weakness = 1 point
- objective oropharyngeal dysphagia (by swallowing studies) = 1 point
- serum CK over 4000 UI/L (yet ≤ 8000 UI/L) at initiation of treatment = 1 point
- CK over 8000 UI/L at initiation of treatment = 2 points.

For descriptive purposes, an anti-HMGCR myopathy with a severity score of ≥ 3 was considered **severe** and < 3 **non severe**.
