# Supplementary material for: Statin-induced anti-HMGCR myopathy: successful therapeutic strategies for corticosteroid-free remission in 55 patients
Source: Arthritis Res Ther. 2020 Jan 8;22:5. doi: 10.1186/s13075-019-2093-6 (PMC6950801; doi:10.1186/s13075-019-2093-6)
Supplement: Supplementary file 2 — Additional file 2 : Table S2. Identifying therapeutic subgroups within the STATIN-PHESEMO study. [file 13075_2019_2093_MOESM2_ESM.docx]

**Supplementary Table S2**

**Identifying therapeutic subgroups within the STATIN-PHESEMO study**

When defined by the initial induction strategy, the STATIN-PHESEMO study included:

1) a **corticosteroid-free induction cohort** and 2) a **corticosteroid-based induction cohort:**

1. in the **corticosteroid-free induction cohort**, two subgroups were seen, as SSI combinations were not used as the initial induction strategy but as a step-up induction strategy:

- the **Solo SSI induction cohort**, i.e. patients initially treated with an SSI alone;
- the **Dual IVIG/SSI induction cohort**, i.e. patients initially treated with IVIG and an SSI; patients failing a solo SSI induction strategy who received a step-up induction IVIG are included in this cohort;

1. in the **corticosteroid-based induction cohort**, two subgroups were seen, as SSI combinations were not used as the initial induction strategy, but as a step-up induction strategy:

- **Dual steroid/SSI cohort**, i.e. patients initially treated with steroids and an SSI;
- **Triple steroid/IVIG/SSI cohort**, i.e. patients initially treated with steroids, IVIG and an SSI; patients failing a dual steroid/SSI induction strategy who received rescue IVIG are included in the Triple steroid/IVIG/SSI induction cohort.

Last, when defined by the muscle strength at both presentation and treatment of anti-HMGCR myopathy, three mutually exclusive, strength-based, cohorts could be identified:

- **early treatment cohort**, i.e. patients treated when strength was still normal;
- **delayed treatment cohort**, i.e. patients presenting with normal strength but treated when proximal weakness ensued;
- **immediate treatment cohort**, i.e. patients presenting with proximal weakness and treated.
