# Supplementary material for: Statin-induced anti-HMGCR myopathy: successful therapeutic strategies for corticosteroid-free remission in 55 patients
Source: Arthritis Res Ther. 2020 Jan 8;22:5. doi: 10.1186/s13075-019-2093-6 (PMC6950801; doi:10.1186/s13075-019-2093-6)
Supplement: Supplementary file 3 — Additional file 3 : Table S3. Chronology of events leading to corticosteroid-free treatment of patients with anti-HMGCR myopathy (N = 14). [file 13075_2019_2093_MOESM3_ESM.docx]

# Supplementary Table S3 Chronology of events leading to corticosteroid-free treatment of patients with anti-HMGCR

# myopathy (*N* = 14)

| **Patient No** | **Age**  **years** | **Sex** | **First serum CK**  **> 500 UI/L** | **Weakness at first CK > 500 UI/L** | **Delay between first increased CK and treatment, months** | **CK decreased by ≥ 50% on discontinuation of statin (UI/L)** | **CK level**  **at treatment onset, UI/L** | **Weakness**  **at treatment onset** |
| --- | --- | --- | --- | --- | --- | --- | --- | --- |
| **1** | 67.7 | F | 1143 | No | 5.0 | No | 1014 | No |
| **25** | 56.7 | M | 1256 | No | 15.0 | No | 886 | No |
| **28** | 72.9 | M | 1517 | No | 78.4 | Yes (375) | 2684 | No |
| **50** | 62.1 | M | 1751 | No | 4.5 | Yes (554) | 554 | No |
| **29** | 60.5 | M | 1876 | No | 9.7 | No | 8177 | No |
| **20** | 76.7 | M | 2925 | No | 57 | Yes (429) | 2124 | Yes |
| **5** | 48.5 | M | 2311 | Yes | 6.4 | No | 1720 | Yes |
| **6** | 65.2 | F | 3128 | Yes | 5.8 | No | 3257 | Yes |
| **2** | 86.1 | M | 2073 | Yes | 12.0 | Yes (1021) | 1363 | Yes |
| **7** | 59.4 | F | 3364 | Yes | 10.0 | Yes (980) | 1533 | Yes |
| **23** | 58.7 | F | 10437 | Yes | 1.0 | No | 10437 | Yes |
| **30** | 48.5 | M | 6000 | Yes | 0 | No | 6000 | Yes |
| **52** | 65.1 | M | 5600 | Yes | 0 | No | 5600 | Yes |
| **54** | 82.2 | M | 2363 | Yes | 0 | No | 2363 | Yes |
