# Supplementary material for: Statin-induced anti-HMGCR myopathy: successful therapeutic strategies for corticosteroid-free remission in 55 patients
Source: Arthritis Res Ther. 2020 Jan 8;22:5. doi: 10.1186/s13075-019-2093-6 (PMC6950801; doi:10.1186/s13075-019-2093-6)
Supplement: Supplementary file 4 — Additional file 4 : Table S4. Corticosteroid-free induction and maintenance therapy of patients with anti-HMGCR myopathy (N = 14) [file 13075_2019_2093_MOESM4_ESM.docx]

**Supplementary Table S4 Corticosteroid-free induction and maintenance therapy of patients with anti-HMGCR myopathy (*N* = 14)**

| **Patient**  **No** | **Severity score** | **Successful induction** | **Failed induction** | **Time to remission**  **months** | **Successful maintenance** | **Follow-up months** | **Weakness at last follow-up** |
| --- | --- | --- | --- | --- | --- | --- | --- |
| **1** | 0 | MTX***** | None | 3.8 | MTX | 40.1 | No |
| **25** | 1 | MTX | None | 4.6 | MTX | 36.2 | No |
| **28** | 0 | MTX | None | 34.0 | Not evaluable (MTX ongoing) | 35.0 | No |
| **50** | 1 | MTX | None | 1.9 | MTX | 16.5 | No |
| **29** | 3 | MTX + AZA/ALLO + IVIG | MTX+IVG | 16.0 | MTX + AZA/ALLO + IVIG | 29.0 | No |
| **20** | 1 | MTX | None | 13.0 | Not evaluable (patient died) | 62.2 | Yes |
| **5** | 2 | MTX | None | 6.4 | MTX | 37.3 | No |
| **6** | 1 | MTX + AZA | AZA | 10.2 | MTX + AZA | 36.5 | No |
| **2** | 1 | MTX + IVIG | None | 1.0 | MTX | 51.1 | No |
| **7** | 2 | MTX + AZA + IVIG | MTX; MTX + AZA | 20.7 | MTX + IVIG | 42.6 | No |
| **23** | 4 | MTX + IVIG | None | 5.0 | MTX | 44.2 | No |
| **30** | 3 | MTX + AZA + IVIG | MTX+IVIG | 9.4 | MTX + AZA + IVIG | 22.0 | No |
| **52** | 2 | MTX + IVIG | None | 3.0 | MTX | 18.0 | No |
| **54** | 1 | MTX + IVIG | None | 2.5 | Not evaluable (MTX + IVIG ongoing) | 10.8 | No |

***** ALLO: allopurinol; AZA: azathioprine; IVIG: intravenous immunoglobulins; MTX: methotrexate.
