# Supplementary material for: Statin-induced anti-HMGCR myopathy: successful therapeutic strategies for corticosteroid-free remission in 55 patients
Source: Arthritis Res Ther. 2020 Jan 8;22:5. doi: 10.1186/s13075-019-2093-6 (PMC6950801; doi:10.1186/s13075-019-2093-6)
Supplement: Supplementary file 5 — Additional file 5 : Table S5. Chronology of events, from disease onset to maintenance therapy, in patients with anti-HMGCR myopathy presenting with normal strength (N = 22) [file 13075_2019_2093_MOESM5_ESM.docx]

# Supplementary Table S5  Chronology of events, from disease onset to maintenance therapy, in patients with anti-HMGCR myopathy

# presenting with normal strength (*N* = 22)

| **Patient No** | **Weakness at treatment** | **Sex** | **Age**  years | **First increased serum CK**  (range) UI/L | **CK decreased by ≥ 50% off treatment after discontinuation of statin** (UI/L) | **Time from first increased CK to treatment**  months | **CK at treatment** UI/L | **Induction with cortico- steroids** | **Induction with IVIG** | **Maintenance treatment**  **for remission** | **Weakness**  **at last**  **follow-up** |
| --- | --- | --- | --- | --- | --- | --- | --- | --- | --- | --- | --- |
| **1** | No | F | 67.7 | 1143 | No | 5.0 | 1014 | No | No | MTX***** | No |
| **24** | No | F | 57.8 | 1501 | No | 13.4 | 6660 | Yes | No | MTX | No |
| **25** | No | M | 56.7 | 1256 | No | 15.0 | 886 | No | No | MTX | No |
| **28** | No | M | 72.9 | 1517 | Yes (375) | 78.4 | 2684 | No | No | Not evaluable | No |
| **29** | No | M | 60.5 | 1876 | No | 9.7 | 8177 | No | Yes | MTX + AZA/ALLO + IVIG | No |
| **34** | No | M | 73.0 | 617 | No | 79.1 | 2832 | Yes | Nos | Not evaluable | No |
| **46** | No | M | 46.5 | 2173 | No | 40.8 | 13339 | Yes | Yes | Unsuccessful | No |
| **50** | No | M | 62.1 | 1751 | Yes (554) | 4.5 | 554 | No | No | MTX | No |
| **55** | No | F | 59.9 | 1030 | No | 2.6 | 696 | Yes | No | MMF | No |
| **Early treatment cohort** n (% or range) | **9** | **F/M 3/6** | **60.5 (46.5-73)** | **1501 (617-2173)** | **Yes 2 (22)** | **13.4 (2.6-78.4)** | **2684 (554-13339)** | **4 (44)** | **2 (22)** | **Successful maintenance with SSI monotherapy 5 (56)** | **0** |
| **4** | Yes | F | 59.1 | 1806 | No | 42.2 | 8390 | Yes | Yes | Unsuccessful | No |
| **11** | Yes | F | 74.3 | 582 | No | 95.0 | 2770 | Yes | Yes | MTX + IVIG | Yes |
| **12** | Yes | M | 69.4 | 3539 | No | 14.0 | 4750 | Yes | No | MTX + IVIG | No |
| **14** | Yes | M | 44.0 | 5613 | No | 11.0 | 11590 | Yes | Yes | MMF + ABA + IVIG | No |
| **15** | Yes | M | 66.2 | 1454 | Yes (702) | 26.0 | 8300 | Yes | Yes | MTX + AZA | Yes |
| **16** | Yes | M | 70.2 | 500 | No | 17.0 | 11755 | Yes | Yes | MTX + AZA | No |
| **18** | Yes | M | 67.5 | 1700 | No | 7.0 | 6400 | Yes | Yes | MTX | No |
| **20** | Yes | M | 76.7 | 2925 | Yes (429) | 57.0 | 2124 | No | No | Not evaluable | Yes |
| **32** | Yes | M | 72.5 | 1202 | No | 24.9 | 4917 | Yes | No | MTX | No |
| **35** | Yes | M | 70.5 | 3064 | Yes (1242) | 19.5 | 3573 | Yes | No | AZA | No |
| **37** | Yes | F | 58.3 | 940 | No | 21.6 | 7804 | Yes | No | MTX | No |
| **38** | Yes | F | 56.7 | 974 | No | 23.1 | 14098 | Yes | Yes | MMF + IVIG | Yes |
| **48** | Yes | M | 63.3 | 3873 | Yes (1838) | 13.4 | 1556 | Yes | Yes | MTX + MMF | No |
| **Delayed treatment cohort,** n (% or range) | **13** | **F/M : 4/9** | **67.5 (44-76.7)** | **1700 (500-5613)** | **Yes 4 (31)** | **21.6 (7.0-95.0)** | **6400 (1556-14098)** | **12 (92)** | **8 (62)** | **Successful maintenance with SSI monotherapy 4 (31)** | **4 (31)** |

*****AZA: azathioprine; IVIG: intravenous immunoglobulins; MMF: mycophenolate mofetil; MTX: methotrexate; SSI: steroid-sparing immunosuppressant.
